# *Angelica gigas* Nakai (Korean Dang-gui) Root Alcoholic Extracts in Health Promotion and Disease Therapy – active Phytochemicals and *In Vivo* Molecular Targets

**DOI:** 10.1007/s11095-024-03809-9

**Published:** 2025-01-08

**Authors:** Junxuan Lü, Cheng Jiang, Joseph J. Drabick, Monika Joshi, Stuthi Perimbeti

**Affiliations:** 1 Department of Pharmacology, Pennsylvania State University College of Medicine, Hershey, PA 17033 USA; 2 Penn State Cancer Institute, Pennsylvania State University, Hershey, PA 17033 USA; 3 Center for Cannabis and Natural Product Pharmaceutics, Pennsylvania State University College of Medicine, Hershey, PA 17033 USA; 4 Department of Medicine Division of Hematology and Oncology, Pennsylvania State University College of Medicine, Hershey, PA 17033 USA

**Keywords:** decursin, decursinol, decursinol angelate, nodakenin, ROCK1/2

## Abstract

*Angelica gigas* Nakai (AGN) root is a medicinal herbal widely used in traditional medicine in Korea. AGN root ethanolic extracts have been marketed as dietary supplements in the United States for memory health and pain management. We have recently reviewed the pharmacokinetics (PK) and first-pass hepatic metabolism of ingested AGN supplements in humans for the signature pyranocoumarins decursin (D, *C*_*max*_ 1x), decursinol angelate (DA, *C*_*max*_ ~ 10x) and their common botanical precursor and hepatic metabolite decursinol (DOH, *C*_*max*_ ~ 1000x). Here we update *in vivo* medicinal activities of AGN and/or its pyranocoumarins and furanocoumarin nodakenin in cancer, pain, memory loss, cerebral ischemia reperfusion stroke, metabolic syndrome and vascular endothelial dysfunctions, anxiety, sleep disorder, epilepsy, inflammatory bowel disease, osteoporosis and osteoarthritis. Given their polypharmacology nature, the pertinent mechanisms of action are likely misrepresented by many cell culture studies that did not consider the drug metabolism knowledge. We report here *Rho*-associated protein kinases (ROCK1/2) as novel targets for DA and DOH. Combining with published inhibitory activity of DOH on acetylcholinesterase, agonist activity of DOH and antagonist/degrader activity of DA/D on androgen and estrogen receptors, D/DA promoting activity for glutamic acid decarboxylase (GAD)- gamma-aminobutyric acid (GABA) inhibitory axis and inhibition of glutamate dehydrogenase (GDH), monoamine oxidase-A (MAO-A) and transient receptor potential vanilloid 1 (TRPV1), we postulate their contributions to neuro-cognitive, metabolic, oncologic, vascular and other beneficial bioactivities of AGN extracts. A clinical trial is being planned for an AGN extract to manage side effects of androgen deprivation therapy in prostate cancer patients.

**Supplementary Information:**

The online version contains supplementary material available at 10.1007/s11095-024-03809-9.

## Introduction

**Fig. 1:**
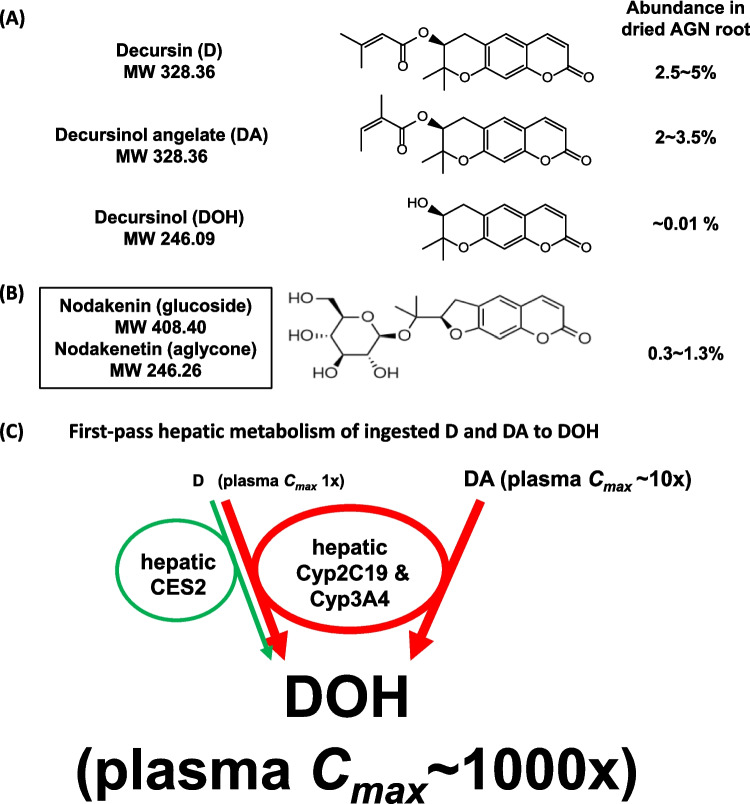
Chemical structures of signature *Angelica gigas* Nakai (AGN) root phytochemicals in alcoholic extracts with documented *in vivo* bioactivities and current knowledge of human metabolism of ingested pyranocoumarins. (**a)** pyranocoumarins decursin (D), decursinol angelate (DA) and their botanical synthesis precursor decursinol (DOH). (**b)** Furanocoumarin glucoside nodakenin and its aglycone nodakenetin. The glucosyl moiety on nodakenin renders it also extractable by aqueous solvents. (**c)** First pass hepatic metabolism of ingested D and DA through portal vein entry to DOH by cytochrome p450 (Cyp) 2C19 and 3A4 isoforms, with additional participation of hepatic carboxyesterase 2 (CES2) for D. The relative peak circulating level for D is ~ 1/10^th^ of DA and ~ 1/1000^th^ of DOH in human blood. Rats and mice follow similar circulating level differentials as in humans. For a full list of phytochemicals identified from AGN, please refer to He, *et al*. [22].

**Fig. 2:**
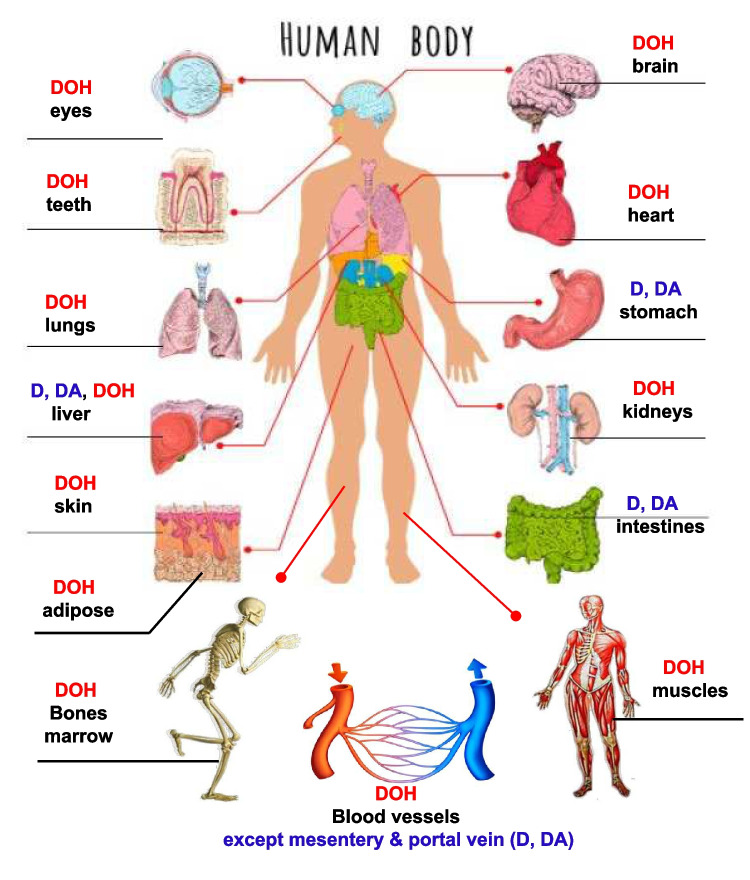
Projected organ/tissue distribution patterns of pyranocoumarins decusin (D), decursinol angelate (DA) and decursinol (DOH) from an orally ingested AGN ethanol extract supplement. The predominant form(s) is labeled for each organ.

**Fig. 3:**
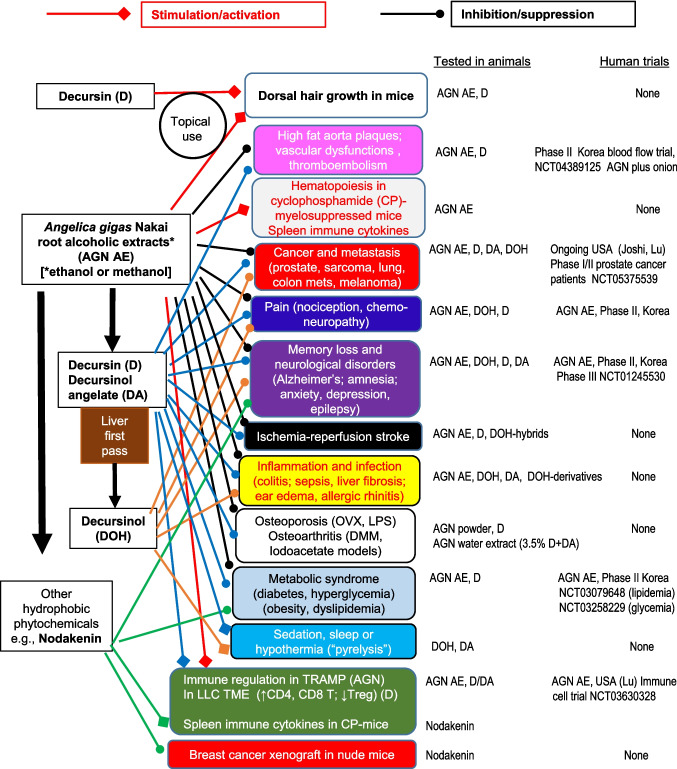
Schematic summary of reported bioactivities of *Angelica gigas* Nakai (AGN) alcoholic extracts and their best known pyranocoumarins (D, DA and DOH) and furanocoumarin glycoside nodakenin in animal models. Lines with diamond ends show stimulatory/activation bioactivity. Lines with bulb ends show inhibitory/suppression bioactivity. A solid line indicates an AGN entity has been tested for at least one indicated bioactivity category in an animal model. Human clinical trial information was identified by registration number at Clinicaltrials.gov site, when available. Modified and updated from Lu, *et al*., *Am J Chin Med*, 2022 [1].

*Angelica gigas* Nakai (AGN) (commonly known as Korean angelica or Korean dang-gui) root is a medicinal herbal widely used in traditional medicine in Korea. AGN root ethanolic extracts have been marketed as dietary supplements in the United States for memory health and pain management. We reviewed in 2022 [1] (https://doi.org/10.1142/S0192415X2250063X) the pharmacokinetics (PK) and metabolism, in humans and rodent models, of AGN signature pyranocoumarin phytochemicals decursin (D), decursinol angelate (DA) and their common botanical precursor decursinol (DOH) (Fig. 1A, C) and summarized the reported *in vivo* medicinal activities of AGN and/or pyranocoumarins on cancer, pain, cognitive disorder/memory loss, cerebral ischemia/stroke, anxiety, sleep disorder, epilepsy, inflammatory bowel disease, sepsis, metabolic disorders, osteoporosis, osteoarthritis, and sperm death. Here, we present the predicted tissue distribution patterns of pyranocoumarins based on their metabolism knowledge (Fig. 2), and we provide an update of newer findings of *in vivo* bioactivities of AGN alcoholic extracts and phytochemicals (summarized in Fig. 3) since the 2022 review, which should be read along with the current work for essential details. In spite of their presumptive “active chemicals” roles, there was a lack of solid experimental data in animal models to systematically delineate the contribution of these pyranocoumarins or other hydrophobic phytochemicals, such as nodakenin (Fig. 1B) to these AGN bioactivities. Further rigorous and comprehensive preclinical research is sorely needed to fill in the knowledge gaps.

Given the current knowledge of the first-pass hepatic metabolite of D and DA to DOH (Fig. 1C), resulting in their respective circulating peak concentration (*C*_*max*_) and area under the curve (*AUC*) in the order of 1 x, 10 × and 1000 × after a single ingested dose of AGN supplement in humans [2, 3] and their likely organ/tissue distribution patterns (Fig. 2), it would be logical to hypothesize that DOH either alone or with its pro-drugs D and DA that had escaped the liver hydrolytic actions, might have mediated many of the reported extra-hepatic beneficial bioactivities in preclinical animal models. Ironically, many cell culture studies have investigated D or DA since the publication of our human PK study [2, 3], without due consideration of the drug metabolism knowledge. Such *in vitro* studies have purported to “have revealed” molecular targets and signaling pathways of D or DA in different cell types. Unfortunately, the results and conclusions of these cell culture studies can be mis-leading and irrelevant to account for the *in vivo* mechanisms of action underlying the beneficial bioactivities of AGN taken orally.

**Fig. 4:**
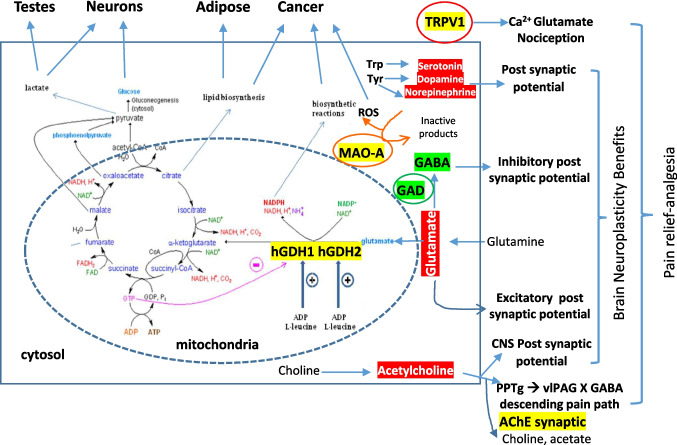
Molecular targets of pyranocoumarins in neuro-cognitive health and pain killing, metabolic homeostasis as well as anti-cancer activity. Yellow shade highlights inhibitory action by AGN pyranocoumarins; Green shade indicates enhancement action. AChE, Acetylcholinesterase; GAD, glutamic acid decarboxylase; GABA, Gamma-aminobutyric acid; GDH/GLUD, glutamate dehydrogenase; MAO-A, monoamine oxidase type A; TRPV1, Transient receptor potential vanilloid 1; a.k.a. capsaicin receptor, vanilloid receptor; vlPAG, ventrolateral periaqueductal gray; PPTg, pedunculopontine tegmentum. Metabolic regulation of hGDH1 and hGDH2 in TCA (tricarboxylic acids) cycle was adapted from Smith *et al*. 2019 [80].

**Fig. 5:**
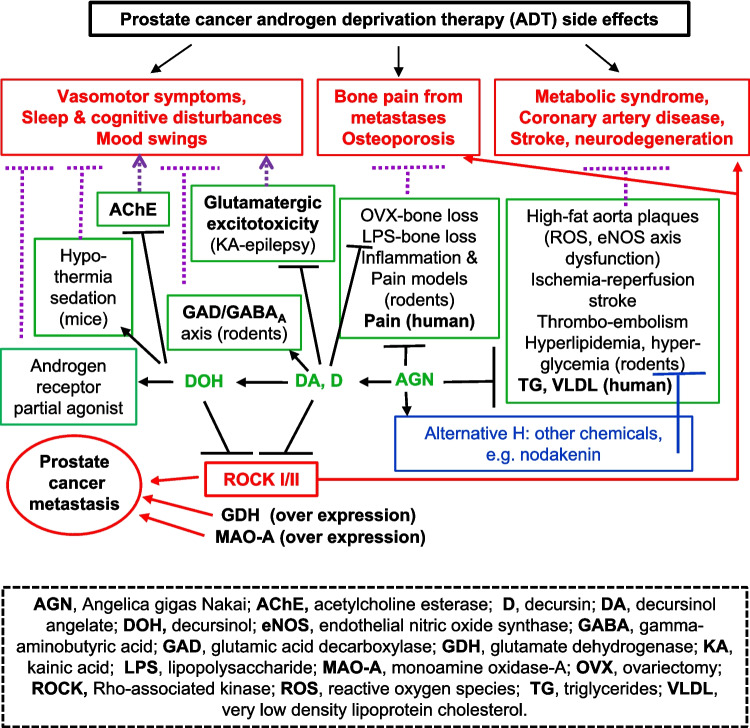
“Mechanistic” connections for AGN ethanol extract to manage side effects of androgen deprivation therapy (ADT) in prostate cancer patients. Key relevant biochemical, cell culture findings, animal models and human trials (when available) are noted. Recent publications in animal studies support the additional contribution from nodakenin to one or more of the health benefit domains.

Herbal natural products like AGN extracts most probably are polypharmacologic in nature, i.e., multiple phytochemicals and multiple targets. The true AGN active phytochemicals and their relevant *in vivo* molecular pharmacodynamic (PD) targets and mechanisms of action are still incompletely understood. To this end, we have, with two independent kinome screening platforms, identified *Rho*-associated protein kinase (ROCK1/2) enzymes as direct targets for DA and DOH (Table I). In addition to our discovery of D and DA as potent novel androgen receptor (AR) and estrogen receptor alpha (ERα) antagonist and degrader and a weak agonist role of DOH for both receptors [4–6], literature review has documented direct inhibitory activity of DOH on acetylcholinesterase (AChE) [7], and D or DA as an enhancer/agonist for glutamic acid decarboxylase (GAD)-gamma-aminobutyric acid A receptor (GABA_A_) inhibitory axis [8], an inhibitor for monoamine oxidase A (MAO-A) [9] and an inhibitor of glutamate dehydrogenase (GDH or GLUD) [10] and as an antagonist of transient receptor potential vanilloid 1 (TRPV1), a.k.a. capsaicin receptor [11]. Based on the known roles of AChE/cholinergic, GABAergic, glutamatergic (~ 80% CNS neurons), monoamine neurotransmitters and TRPV1/Ca and ROCK1/2 in neural functions, neuronal metabolism, death and vascular dysfunctions in addition to oncogenesis, we integrate these enzymes and receptors for mediating the various beneficial neuro, vascular and metabolic activities of pyranocoumarins and AGN extracts (Fig. 4). Beside the pyranocoumarins, the furanocoumarin nodakenin (Fig. 1B) has emerged as another AGN phytochemical that likely contributes to some of the reported protective bioactivities, including against memory loss, hyperlipidemia, bone loss, inflammation, breast cancer growth and myelosuppression (Fig. 3) [12–16, 17]. We advocate human clinical trial research for managing and treating side effects of androgen deprivation therapy (ADT) for prostate cancer patients as a reasonable clinical application of the mechanistic knowledge (Fig. 5).

**Table 1:** The DiscoveRX KINOMEscan and ReactionBiology Profiling of Decursinol (DOH) and Decurisnol Angelate (DA) Effects On Protein Kinases in Comparison to Reported Inhibitory Potency Against Other Non-kinase Enzymes or Sex Hormone Receptors

Testers	Eurofins DiscoveRx		Reaction Biology, Inc		Kang *et al*. 2001 [7]	Guo *et al*. 2007 [5]; Jiang *et al*. 2007 [6]	Lee *et al*. 2017 [9]	Chang *et al*. 2023 [10]
Enzyme	ROCK1 *Kd*, µM	ROCK2 *Kd*, µM	ROCK1 *IC*_*50*_, µM	ROCK2 *IC*_*50*_, µM	AChE^1^ inhibition *IC*_*50*_, µM	AR^2^; ERα *IC*_*50*_, *EC*_*50*_, µM	MAO-A^3^ inhibition *IC*_*50*_, µM	GDH^4^ inhibition *IC*_*50*_, µM
Compound								
DOH	7.5	14.5	81	85	28	(Agonist *EC*_50_ ~ 10)	> 10	Not tested
DA	2.1	5.6	18	23	Not tested	*IC*_*50*_ ~ 5	12.8	1.4
D	Not tested	Not tested	Not tested	Not tested	390	*IC*_*50*_ ~ 5	1.89 (*Ki* 0.17)	1.0
Nodakenin	Not tested	Not tested	Not tested	Not tested	68	Not tested	Not tested	Not tested

^1^AChE, acetylcholine esterase; ^2^AR, androgen receptor; ERα, estrogen receptor α; ^3^MAO-A, monoamine oxidase A; ^4^GDH/GLUD, glutamate dehydrogenase

## Phytochemicals in AGN Alcoholic Extracts

Figure 1A shows the best-known signature pyranocoumarins D and DA (two isomers on the side chain) in the ethanolic or methanolic extracts of dried AGN roots. The content of D is usually higher than DA [1, 18]. Depending on the extraction protocols and sources of the AGN roots, their combined yields can range from single to low double digit % on a dry root basis [19, 20]. These compounds are absent from other major Oriental medicinal *Angelica* species such as *A. sinensis* (Chinese Dang-gui), and *A. acutiloba* (Japanese). Their botanical synthesis precursor DOH is about 2 to 3 orders of magnitude less abundant than D or DA in the dried root. These pyranocoumarins are hydrophobic in nature and not extractable by boiling in water, which is the most common traditional herbal medicine preparation method.

Figure 1B shows the structure of furanocoumarin nodakenin (glucoside) and its aglycone nodakenetin. The presence of the glucose moiety in nodakenin makes it readily extractable in aqueous extraction preparations, as in the case of a menopausal symptom relief herbal preparation marketed as EstroG-100 that is based on hot water extraction of AGN plus *Cynanchum wilfordii* and *Phlomis umbrosa* [21]. In such products, nodakenin is used as the marker compound for AGN. Nodakenin has been reported for memory benefit, lipid lowering activity, breast cancer xenograft growth inhibition, bone loss prevention, anti-inflammation, as well as hematopoiesis in animal models [12–17].

Beside these better studied compounds, there are more than 50 other phytochemicals identified in AGN roots, including simple coumarins, furanocoumarins, pyranocoumarins, volatile oils and flavonoids. For details of their structures, please see review by He, *et al*. [22].

## Current Knowledge of Pharmacokinetics and Metabolism of Major Pyranocoumarins and Nodakenin

While the topic of D and DA PK and metabolism has been reviewed in depth previously [1, 23], Fig. 1C provides a graphic summary of the first pass hepatic metabolism of the ingested D and DA from AGN extract or by *ip* injection delivery in rodent models. These pyranocoumarins are absorbed from the intestine or mesentery peritoneum and delivered to the liver through the portal vein system. In 10 healthy men and 10 women, we tested the single oral dose PK metrics for D and DA and DOH after they had ingested 800 mg of INM176 AGN [2]. Plasma mean time to peak *t*_*max*_ was 2.1, 2.4 and 3.3 h and mean peak concentration *C*_*max*_ was 5.3, 48.1 and 2,480 nmol/L for D, DA and DOH, respectively,mean area under the curve *AUC*_0–48h_ of 37, 335 and 27,579 h∙nmol/L, revealing the orders of magnitude differences in their relative abundance in the blood circulation. The terminal elimination half-life (*t*_*1/2*_) for D and DA of 17.4 and 19.3 h was longer than that of DOH of 7.4 h. The human data supported an extensive conversion of D and DA to DOH (*AUC*_DOH_/*AUC*_total_ = 98.7%) at the dietary supplement dose tested, consistent with rodent PK models [24–27]. The Cyp isoforms of CYP2C19 and 3A4 were identified by us as the major human isoforms for D and DA metabolism to DOH, whereas carboxyesterase-2 (CES2) further contributed to metabolizing D, but not DA, to DOH [3]. Based on these knowledges, we project the predominant distribution patterns of D, DA and DOH in human organs/tissues in Fig. 2.

Our 2022 review [1] questioned the authenticity of the “AGN” for a PK study in healthy men reported by a Korean group [28]. Concisely, their 4.6 g of “AGN” root powder per subject contained only 0.055 mg of D, 0.184 mg of DA [28], in contrast to our subjects each taking in 119 mg D and 77 mg DA [2]. They reported *t*_*max*_ values for D, DA and DOH of 0.44, 0.31 and 0.64 h, respectively, much sooner than our findings. The *t*_*1/2*_ estimates for D, DA, and DOH (3.03, 4.04, and 2.62 h, respectively) were much shorter than our reported values above. They reported *C*_*max*_ for D and DA and DOH of 11.87, 7.72 and 0.92 ng/ml, respectively. The *C*_*max*_ for D would be impossible with a D intake of 0.055 mg because a *C*_*0*_ of 5–6 ng/ml could be calculated if the dose were given by *iv* injection to a human weighing 80 kg, i.e., ~ 10 L blood volume. These discrepancies from our PK metrics might have arisen from the authenticity and physical states (root powder vs. extracted chemicals) of the AGN and the vast differences in the dosage of D and DA.

Nevertheless, this Korean study provided PK metrics for nodakenin in humans when the “AGN” powder dose provided 1.095 mg of nodakenin with *C*_*max*_ of 0.95 ng/ml [28]. The *t*_*1/2*_ for nodakenin was 6.28 h with a very large apparent volume of distribution *V*_*d*_ 2800 L. In a rat study, nodakenin *t*_*1/2*_ of 3.3 h was reported after an *iv* dose [29]. In an earlier rat study after oral administration of an herbal mix containing nodakenin and D/DA, much longer *T*_*max*_ was reported for nodakenin than DOH [30], such that nodakenin *t*_*1/2*_ was 4.6 and 6.2 h for 2.5 and 10 mg/kg doses of the herbal mix. Interestingly, in spite of very low abundance of its aglycone nodakenetin in the herbal mix, comparable *T*_*max*_ and *C*_*max*_ were observed for nodakenin and nodakenetin in the rat blood [30], supporting an *in vivo* modest deglucosylation metabolism of nodakenin to nodakenetin. Validation of this metabolic relationship in humans is needed.

## Updated *In Vivo* Bioactivities of AGN Extracts and Phytochemical Compounds Through Alimentary or Intraperitoneal Delivery

Since AGN root powder and its alcoholic extracts are researched and developed toward eventual human applications for health promotion as oral herbal supplements or for disease treatment as oral drugs, we therefore update on the bioactivities reported in animal models using gavage delivery or intraperitoneal (*ip*) injection. The *ip* route has been well validated in rodent models to simulate oral drug absorption and transport through the portal vein system to the liver for first-pass metabolism [31]. Figure 3 provides a graphic summary of up to date reported bioactivities of AGN root powder and alcoholic extracts in animal models and human studies. A recent study of topical application AGN or D in promoting hair growth [32] was included to highlight applicability of parenteral delivery to expand their uses in health promotion and disease therapy. Please refer to our 2022 review [1] for details for most of the covered bioactivities. The following sections update on publications since our 2022 review in key health domains.

### Anti-cancer Activities—an Update on Nodakenin

The broad spectrum anti-cancer activities of AGN extracts and pyranocoumarins in organ sites including prostate, lung, colons have been extensively reviewed and summarized in our 2022 review [1]. Since then, a single author study by Tae Woo Kim [14] reported in 2023 that nodakenin administered *ip* at 10 and 30 mg/kg twice weekly inhibited the growth of MDA-MB231 *sc* breast cancer xenograft dose-dependently (~ 88% inhibition at the higher dose) in a 5-week *in vivo* assay, without any negative impact on the body weight of the animals as a measure of their well-being. Using cell culture models, Kim found that nodakenin, in a concentration-dependent manner, inhibited breast cancer cell viability by induction of caspase-3-dependent apoptosis which was attenuated by the caspase inhibitor z-VAD-FMK. The cell death involved PERK-mediated signaling pathway and calcium (Ca^2+^) release, endoplasmic reticulum (ER) stress and reactive oxygen species (ROS) activating the Nox4 axis. Nodakenin also suppressed epithelial–mesenchymal transition (EMT) phenotypes, a key feature of metastatic cancer. This study supports the polypharmacology hypothesis that additional phytochemicals beyond pyranocoumarins contribute to the anti-cancer activities of AGN, consistent with our earlier findings in TRAMP prostate cancer model that equal molar D/DA was inferior to AGN at inhibiting the growth of aggressive neuroendocrine carcinoma (NECa) and at supporting the survival of NECa-bearing mice [33] and a narrower range of molecular hubs and networks affected in the prostatic epithelial lesions and NECa by D/DA than by AGN [33, 34].

### Pain Killing Effects and Further Mechanistic Insights

Following the initial demonstration of dose-dependent anti-nociceptive activities in mouse models for AGN extract [35], the same group reported the efficacy and mechanisms of orally-administered DOH (5 to 200 mg/kg body weight) in mice [36]. Their results indicated that gavaged DOH exerted dose-dependent anti-nociceptive efficacy in the various acute pain models (ED_50_ ~ 50 mg/kg, peak time 30 min post dose). In nociceptive models induced by footpad intraplantar injection of formalin and by intrathecal (*it*) injection of cytokines or irritants such as TNF-α, IL-1β, IFN-γ, substance P or glutamate, DOH dose-dependently decreased pain responses. Studies with receptor antagonists suggested DOH might involve noradrenergic, serotonergic, adenosine A2, histamine H1 and H2 receptors, with exception of the opioid receptor signaling pathway [36].

In the spirit of research rigor and reproducibility, we and collaborators [37] have 1) assessed tolerance development to the antinociceptive effects of once-daily *ip* DOH (50 mg/kg) in mouse acute thermal pain models, 2) tested anti-allodynic efficacy and tolerance in a model of chemotherapy(cisplatin)-induced neuropathic pain (CINP) and 3) probed the involvement of select receptors in mediating the pain-relieving effects with antagonists. Our results replicated antinociception effects of DOH in both the hot plate and tail-flick assays and reversed the mechanical allodynia of mice with cisplatin-neuropathy (per Von Fry test). Tolerance was gradually detected to the antinociceptive effects of DOH in the hot plate and tail-flick assays and in the CINP mice (~ 10 days), but slower than the rapid tolerance development for the prototypic opioid morphine (< 3 days). Pretreatment with 5-HT_2_ antagonist methysergide, 5-HT_2A_ antagonist volinanserin, or 5-HT_2C_ antagonist SB-242084 did not attenuate DOH antinociception in the tail-flick assay, nor did the cannabinoid inverse agonists rimonabant and SR144528 modify DOH anti-allodynia in CINP mice. Contrary to the Choi study [36], pretreatment with the opioid antagonist naloxone partially attenuated the anti-allodynic effects in CINP mice. Furthermore, our investigation uncovered DOH-dose dependent hypothermia (“pyrolytic”) and sedation (ataxia) effects resembling such known actions of morphine. Therefore, the receptor types involved and mechanisms are likely more complicated than initially reported [36]. The sedation and ataxia could have confounded the interpretation of the initial DOH study [36] and a study by Seo *et al*. of the analgesic effect of DOH using the acetic acid-induced writhing test in male ICR mice [38] in whom only an orally-administered dose of 50 mg/kg was efficacious when the sedation effect was strong, but not at 25 or 10 mg/kg with minimal sedation effect.

In a model of “epidural injection” for pain killing, bypassing the conventional oral herbal supplement approach, an *intrathecal (it)* injection of D was shown to alleviate paclitaxel-CINP in a mouse model [11]. They examined the impact of *it* D (50 mg/kg), (L5-L6 intervertebral space, 5 μL D or vehicle every other day) for 6 days. Mechanical allodynia (per Von Fry test) showed no relief by the first injection of D, but the second and third injections each blunted the allodynia for up to 3 h. Since CINP pathophysiology involves damage to neuronal networks and dysregulation of signal transduction due to abnormal Ca^2+^ levels, the authors used a F11 cell culture model (a somatic cell hybrid of a rat embryonic dorsal root ganglion [DRG] and mouse neuroblastoma cell line N18TG) to test the direct impact of D on capsaicin (hot pepper pain trigger)-induced rise of intracellular Ca^2+^ level and neurite outgrowth. They observed a concentration-dependent response in each metric to D and that D antagonized the transient receptor potential vanilloid 1 (TRPV1)-Ca^2+^ release with IC_50_ ~ 1 µM. A direct *it* injection of DOH and DA should be tested in the same *in vivo* model for structure activity relationship (SAR) to address whether the action was mediated by DOH or other D or DA-mediated mechanism. The SAR study should also be applied to the *in vitro* tests for DOH and DA.

### Cognitive/Memory Health

AGN ethanol extract trade-named INM-176 was the active herbal ingredient for the supplement product Cogni.Q that had been marketed in the US since 2012 by Quality of Life Labs and currently out of stock due to COVID supply chain issues. A phase II clinical trial in elderly Korean patients with Alzheimer’s dementia had been published in Korean language with promising efficacy [39]. However, the results of a follow-up Phase III trial comparing INM176 AGN to donepezil, an AChE inhibitor drug, have not been published (Clinicaltrials.gov NCT01245530). The first human memory clinical trial [39] was based on test tube-based discovery of DOH (not D) as an AChE inhibitor [7] (Table I) and considerable animal model studies. Additional published animal studies provided further support for the cognitive/memory benefit of AGN alcoholic extracts and the pyranocoumarins. No further progress has been reported since our 2022 review [1].

In addition to animal models with the pyranocoumarins consistent with their potential active (prodrug) compound role, nodakenin was also found to ameliorate cholinergic drug scopolamine (1 mg/kg, *ip*)-induced memory disruption in mice in the passive avoidance, the Y-maze, and the Morris water maze tests at 10 mg/kg, *po* [13]. Yet, *in vitro* test showed nodakenin was a rather weak inhibitor of AChE activity (IC_50_ 84.7 µM) [13], consistent with an earlier reported IC_50_ of 68 µM (Table I) [7]. Nevertheless, an ex vivo test found 6 h after dose of nodakenin the brain slice AChE activity was lowered [13], consistent with its slow *T*_*max*_ [30]. These results suggest that nodakenin may contribute to alleviation of cognitive impairment, and that its beneficial effects are mediated, in part, via the enhancement of cholinergic signaling. More rigorous animal model studies for nodakenin, alone and in combination with DOH, D or DA are needed to define the scope and extent of each of these AGN chemicals toward the cognitive benefit domain.

### Additional Neuro-psycho-behavioral Benefits of Pyranocoumarins Suggest Multiple Molecular Targets

As has been reviewed in our 2022 paper [1], epilepsy is a neurological disorder with recurrent unprovoked seizures as the main symptom. D was reported to ameliorate glutamatergic kainic acid (KA)-induced *status epilepticus* in mice [40]. The authors administered D *ip* (20 mg/kg) to male 7-week-old C57BL/6 mice 30 min prior to KA (30 mg/kg, *ip)* and then examined behavioral seizure score, electroencephalogram, seizure-related expressed protein levels, neuronal cell loss, neurodegeneration, and astrogliosis. The D-pretreated KA-injected mice showed decreased behavioral seizure activity and fewer intense and high-frequency seizure discharges in the parietal cortex during 2 h observation compared with the group treated only with KA. Furthermore, their *in vivo* results indicated that D inhibited selective neuronal death, astrogliosis, and oxidative stress induced by KA. DOH and DA should be tested in the same model for SAR.

DA was shown to augment pentobarbital-induced sleeping behaviors involving the inhibitory GABA_A_-ergic systems in mouse and rat models [8]. Oral administration of DA (10, 25 and 50 mg/kg) markedly suppressed spontaneous locomotor activity (sedation) for the mice. DA, in a dose-dependent manner, prolonged sleeping time, and decreased the sleep latency by pentobarbital (42 mg/kg), akin to the impact of the prototypic agonist muscimol and increased the number of sleeping mice. In rats by electroencephalography (EEG) monitoring, DA (50 mg/kg, *po*) modulated sleep architectures and reduced the counts of sleep/wake cycles, increased total sleep time, but not non-rapid eye movement (NREM) and rapid eye movement (REM) sleep. At the molecular/receptor level, DA (0.001, 0.01 and 0.1 µg/ml ≈ 0.003, 0.03 and 0.3 µM) increased intracellular Cl^−^ influx level in cultured rat hypothalamic primary neuronal cells. In addition, DA increased the protein expression of glutamic acid decarboxylase (GAD_65/67_) and GABA_A_ receptor subtypes. Based on metabolism and PK knowledge of D and DA, DOH might have contributed to improving sleep (sedation; locomotor decline) and should be tested side-by-side to address this prediction. The potency of DA (by inference D) to trigger GABA-_A_ Cl^−^ ion channel axis lends credence to probable engagement *in vivo*.

Lee and co-workers [41] examined the effects of AGN (methanol 80%) extract treatment in a rat model of depressive and anxiety-like behaviors, induced by chronic stress hormone corticosterone injections to cause dysregulation in the hypothalamic–pituitary–adrenal (HPA) axis. In their experiment, male rats received 10, 20, or 50 mg/kg AGN *ip* daily at 30 min prior to a daily injection of corticosterone for 21 consecutive days and significantly reversed the depression and anxiety-like behavioral abnormalities in a dose-dependent manner. AGN also blocked the increased tyrosine hydroxylase expression (dopamine-noradrenaline synthesis) in the *locus coeruleus*, and restored expression levels of brain-derived neurotrophic factor (BDNF) and its receptor TrkB mRNAs in the hippocampus. When compared with the anti-depressant drug fluoxetine (Prozac) (a selective serotonin reuptake inhibitor SSRI), AGN extract was fully efficacious but only 1/5 as potent (50 mg AGN/kg ≈ 10 mg/kg fluoxetine) at regulating the above behavioral and biochemical metrics. DOH gavage has been found to increase the serotonin (5-hydroxytryptamine) level in rat brain [42] in that the highest concentration was observed in frontal cortex and striatum at 10 mg DOH/kg body weight. Based on metabolism knowledge of D and DA (Fig. 1C), DOH should be compared side-by-side with these parental drugs to validate the SAR in these neuro-behavior activities.

### Cerebral Ischemic Stroke Prophylaxis and Treatment

As we last reviewed [1], two groups studied the impact of AGN alcoholic extracts on middle carotid artery occlusion (MCAO)-induced stroke in mice [43] and global cerebral ischemia model by bilateral common carotid artery occlusion (BCCAO) in gerbils [44]. In mice [43] in a prophylactic setting, a methanol (99%) extract of AGN root was orally administered twice (day −1 and 1 h) prior to starting a 90-min MCAO. The AGN pre-treatment at 1000 mg/kg per day for two consecutive days decreased the infarct ischemic brain volume by ~ 40% and ameliorated the morphological deteriorations in the brain neuron cells. The neuroprotective mechanism was associated with decreased neuronal death and attenuation of ERK-related signaling pathway in the ipsilateral hippocampus hemisphere in mice and decreased inflammatory cytokine (TNF-α, IL-6). This group performed a follow up study of PK in rats for plasma D concentration, which showed peak absorption around 4 h and rapid elimination (barely detectable after 7 h) after oral administration of AGN at a dose of 100 mg per rat (~ 300 mg/kg bw). Unfortunately, they did not measure DOH or DA.

Another group tested “therapeutic” utility of AGN extract in the gerbil global cerebral ischemia BOCCA model [44]. Starting 30 min following the completion of 5-min BOCCA, the investigators treated the inflicted gerbils orally with AGN at 200, 350 and 400 mg/kg (extracted with 98% ethanol) and 25 mg/kg of D matching that in 350 mg/kg as AGN extract (D ~ 7.3%). They showed that the pyramidal neurons located in the *cornu ammonis* 1 *(CA1)* among the hippocampal subfields were dead at 5 days after the ischemia; however, treatment with the AGN extract (350 and 400 mg, not 200 mg) and D spared the pyramidal neurons from ischemic death. The ischemia breached the blood–brain barrier (BBB) from 2 days after the BOCCA and was attenuated by post-ischemia treatment with efficacious AGN dose or D ( less dye leakage to *CA1* parenchyma). Furthermore, astrocyte endfeet, as a component of the BBB, were severely damaged at 5 days after the ischemia, but were protected by the post-BOCCA treatment with AGN or D. Moreover, AGN or D post-treatment preserved spatial memory in an 8-arm radial maze test (8-ARMT) and learning memory in passive avoidance test (PAT) while the vehicle group incurred memory impairment after 5 days following BOCCA.

In retrospect, earlier studies with 2 DOH hybrid compounds in comparison with DOH or the respective drug counterpart in the gerbil transient cerebral ischemia stroke model [45, 46] are worthy further discussion. In the first study, oral α-lipoic acid (ALA)-DOH hybrid [45] was compared with ALA, or DOH alone against ischemia–reperfusion damage in the gerbil hippocampal *CA1* region induced by 5 min of BOCCA. The 20 mg/kg ALA-DOH hybrid pre-treatment regimen (on day −2, −1 and 30 min before 5-min ischemia) protected the pyramidal neurons from ischemic damage. In addition, 20 mg/kg ALA-DOH pre-treatments markedly decreased the activation of astrocytes and microglia in the same brain region 4 days after ischemic injury. The single drugs at both 10 and 20 mg/kg and the 10 mg/kg ALA-DOH hybrid pre-treated groups showed no neuroprotective effect against ischemic damage 4 days after BOCCA. On the other hand, none of the post-ischemia treatments (30 min after 5-min BOCCA plus an additional dose on day + 1) with each drug alone or the hybrid drug showed any neuroprotective effect against ischemic damage, supporting only a prophylactic utility of the threshold efficacious dose of the hybrid drug.

With the same gerbil (BOCCA for 5 min) model and Sprague–Dawley rats for transient focal cerebral ischemic damage (90 min middle cerebral artery occlusion, MCAO), the second study investigated neuroprotective effects of aspirin (acetyl salicylic acid ASA), DOH and DOH-aspirin hybrid (DOH-ASA) by *ip* injection as either pre-treatment or post-treatment regimen and came to same conclusion of threshold pre-treatment prophylactic efficacy of the hybrid drug and lack of any efficacy in the post-ischemic therapy setting [42]. Notably, in these early studies, DOH failed to protect against ischemia stroke models (gerbils, rats) at the tested dosage of 20 mg/kg either as pre or post-treatment. Whereas D at 25 mg/kg was reported to offer detectable protection in the most recent study in gerbils in post-ischemia treatment context [44]. In these studies of AGN extracts or purified compounds, threshold-dose response appeared to be a common theme. Side-by-side comparison of D, DA vs. DOH with dose titration curves will be necessary to settle the *in vivo* active compound issue for the cerebral stroke protective benefit.

### Metabolic Syndrome, Vascular Dysfunctions and Thromboembolism

Since our last review [1], additional benefits of AGN extract on hyperlipidemia, obesity and vascular dysfunctions have been reported in a rat model [47]. In this latest study, 40 rats on a high fat diet (HFD) were given AGN ethanol extract (~ 13% D) by daily gavage in the dose range of 100–300 mg/kg for 8 weeks. The muscarinic vascular relaxation responses to acetylcholine ex vivo were impaired in HFD rats, while AGN dosing reversed the ex vivo relaxation pattern in a dose-dependent manner. Endothelial dysfunctions, including increased aorta plaque area, reactive oxygen species (ROS), and decreased nitric oxide (NO) and endothelial nitric oxide synthase (eNOS) Ser1177 phosphorylation, were observed in HFD rats, whereas AGN dose-dependently reversed these metrics and the associated biochemical signaling. Furthermore, AGN dosing regulated endoplasmic reticulum (ER) stress and IRE1alpha sulfonation and its subsequent *sirt1* RNA decay through controlling the regulated IRE1alpha-dependent decay (RIDD)-signaling, ultimately promoting NO bioavailability via the SIRT1-eNOS axis in aorta and endothelial cells. As a sensor for low cellular energy status, SIRT1 is a NAD^+^-dependent deacetylase that controls lipid and key metabolic functions by deacetylating target proteins. In HUVEC cells, equi-molar D to AGN extract exhibited a similar effect in alleviating endothelial dysfunctions. These data suggest that AGN or D regulates dyslipidemia-associated vascular dysfunction by controlling ROS-associated ER stress responses, especially IRE1alpha-RIDD/SIRT1 decay and the AMPK-SIRT1 NO axis.

In similar high fat-diet induced obesity mouse model, an earlier study [48] investigated the anti-obesity potential of D using mice fed a high-fat diet (HFD) vs. HFD plus D 200 mg/kg (ppm) for 7 weeks. Mice consuming HFD + D decreased weight gain, blood triglyceride content and total cholesterol content and fat size compared with those that received the HFD alone. Feeding D improved the glucose tolerance in mice and reduced the secretion of HFD-induced adipocytokines leptin, resistin, IL-6 and MCP-1. In mouse 3T3-L1 cells, *in vitr*o D exposure inhibited their differentiation toward adipocytes and the expression of fatty acid synthase. Bae, *et al*. evaluated the antidiabetic and lipid metabolism effect of AGN extract in a type 2 diabetic C57BL/KsJ-db/db mouse model [49]. AGN gavage at 20, and 40 mg/kg for 8 weeks decreased fasting glucose and insulin levels, decreased the areas under the curve of glucose in oral glucose tolerance and insulin tolerance tests. AGN also ameliorated hepatic steatosis, hyperlipidemia, and hypercholesterolemia. Tissue measurements suggested that the glucose-lowering effect of AGN was associated with the activation of liver AMP activated protein kinase (AMPK), Akt, and glycogen synthase kinase-3. AGN was 5 times more potent than first line diabetes drug metformin (100 mg/kg) in regulating these beneficial biochemistry and physiology metrics. The same group explored the impact of AGN on lipid dysregulation in more detail [50] using C57BL6/J mice fed a HFD. Gavage of 10, 20 and 40 mg/kg doses of AGN for 16 weeks attenuated glucose and insulin intolerance, hepatic steatosis and inflammation, and hypertriglyceridemia induced by the HFD. AGN significantly suppressed hepatic de novo lipogenesis in association with an activation of AMPK (cellular energy sensor). In cell culture model, HepG2 cells treated with free fatty acid mixture (oleate:palmitate = 2:1) increased de novo lipogenesis and consequently lipid droplet formation. Addition of D or DA suppressed lipid accumulation in HepG2 cells along with an increased Sirt1 expression. These results pre-saged the metabolic effects of AGN might be linked to the induction of Sirt1 and consequent activation of AMPK. Adding to the complexity, a cell culture study of adipose stem cells (ASCs) exposed to D or DA [51] showed significantly inhibited adipocyte differentiation involving downregulated CCAAT/enhancer binding protein α (C/EBPα), peroxisome proliferator-activated receptor γ (PPARγ), adipocyte fatty acid binding protein (aP2), fatty acid synthase (FAS), and acetyl-CoA carboxylase (ACC) at both mRNA and protein levels. Increased phosphorylation of glycogen synthase kinase (GSK)−3β to slow down β-catenin degradation and increased β-catenin nuclear translocation appeared to play an upstream signaling role for D and DA to mediate the anti-adipogenic effect. Given the portal vein delivery of D and DA to liver (Figs. 1C, & 2) where significant lipid metabolism (cyclic lipogenesis driven by meal-fast cycles) occurs in hepatocytes, D and DA may directly affect these biochemical pathways before their conversion to DOH. SAR studies of DOH along with D or DA are warranted to assess their relative contributions for lipogenesis and glycemia control in the animal models.

AGN ethanolic extract was evaluated for activity against thromboembolism in an experimentally-induced platelet aggregation model [52]. Oral AGN at 40, 80 and 160 mg/kg 1 h before epinephrine and collagen tail vein *iv* injection reduced death or paralysis in mice caused by the collagen/epinephrine-induced thromboembolism in a bell-shaped manner, such that the highest AGN dose had less protection than the middle dose, which was more potent than aspirin. There was a corresponding inhibition of collagen-induced human platelet aggregation in a concentration-dependent manner. Additionally, AGN-treated mice did not show severe gastric ulcerating bleeding compared to aspirin-treated mice. D at 12.5 mg/kg replicated the protective effect of the efficacious dose of AGN. A comparison of the efficacy of DOH with D, DA and AGN will inform the *in vivo* active chemicals to mediate the anti-thromboembolic benefits.

In addition to pyranocoumarins, nodakenin was reported to decrease obesity and its complications *in vivo* [12] in a high fat diet-induced obesity model. Mice were fed a high fat diet for 8 weeks to induce obesity and were given daily oral nodakenin (10 and 20 mg/kg body weight) for 5 more weeks to compare with orlistat (FDA approved drug to decrease fat absorption) at the same dosages. Nodakenin showed comparable potency to orlistat for suppression of weight gain, dyslipidemia and the development of fatty liver in HFD-induced obese mice. In addition, the authors linked inhibition of adipogenic differentiation and obesity-induced inflammation and oxidative stress to the suppression of the VLDLR and MEK/ERK1/2 pathways. In the differentiated 3T3-L1 adipocytes cell culture model, nodakenin regulated adipogenic transcription factors and genes associated with triglyceride synthesis.

Two human trials have been initiated in Korea (NCT03258229; NCT03079648) for assessing the metabolic syndrome benefits of AGN ethanol extracts. Another trial (NCT04389125) has been initiated in Korea to test blood flow vascular function action of AGN plus onion extracts. The outcome of NCT03079648 12-week clinical trial was reported [53], with positive triglyceride and VLDL-cholesterol lowering of 9.4% over baseline in AGN group vs. 12.5% increase in both metrics in the placebo arm. These results suggest that AGN supplement use can help manage or prevent hypertriglyceridemia. Optimization of AGN dosage and assessment of its long-term safety should be further pursued.

### Inflammatory Bowel Diseases (IBD) and Infections

We have extensively reviewed the anti-inflammatory and anti-infection beneficial activities of AGN and pyranocoumarins in our 2022 article [1]. In earlier work, Korean researchers had used dextran sulfate sodium (DSS) induced IBD-UC model to evaluate the effects of AGN ethanol extract in mice [54]. Daily oral AGN at 10, 20, and 40 mg/kg for 7 days alleviated weight loss, decreased disease activity index scores, and reduced colon shortening in mice with DSS-induced UC and the tissue damage at the histology level. At molecular marker level, AGN dosing decreased IL-6 and TNF-α inflammatory cytokines in serum and colon tissue and suppressed the increased expression of COX-2 and HIF-1α and the increased production of protective PGE_2_ in colon tissue. In the same IBD model, another Korean team examined DA for prophylactic/therapeutic activities [55]. DA *ip* injection every other day (0.4 and 4 mg/kg) starting 1 day before DSS exposure attenuated the severity of colitis including a reduction in weight loss, colon shortening dose-dependently, and a protection from colonic tissue damage induced by DSS at the higher dose. DA decreased type 17 helper T (Th17) cells and neutrophils in the colitis tissues. Together, these studies in the DSS-UC IBD models suggest prevention and clinical management by AGN and pyranocoumarins as key chemical mediators.

New progress with nodakenin with respect to IBD-UC models is noteworthy. Using a different chemical to induced UC, a 2024 paper from India researchers [16] evaluated the coloprotective effect of nodakenin and possible involvement of the nuclear factor kappa B (NFƙB), cyclooxygenase-2 (COX-2), inducible nitric oxide (iNOS), nucleotide-binding receptor domain 3 (NLRP3) inflammasome pathway. In mice, UC was induced by 2,4,6-trinitrobenzene sulfonic acid (TNBS). Nodakenin (10, 20, and 40 mg/kg) was given by gavage, and disease activity index (DAI) score and histological score were evaluated. Peroxidation and anti-oxidation activities markers including malondialdehyde (MDA), myeloperoxidase (MPO), superoxide dismutase (SOD), nitric oxide (NO) levels as well as TNF-α, and IL-6 concentration were evaluated in colon homogenate. Nodakenin treatment lowered the DAI score, MPO, MDA, NO and TNF-α, IL-6 levels while elevated SOD levels as compared to the disease control group. Colon sample mRNA expression measurement showed nodakenin (40 mg/kg) modestly downregulated the expression of NFƙB (1.24-fold), iNOS (1.2-fold), COX-2 (1.98-fold), NLRP3 (1.78-fold), IL-1β (1.29-fold), and IL-18 (1.17-fold). Taken together, nodakenin alleviated TNBS-induced colitis by presumably suppressing NFƙB-mediated NLRP3 inflammasome pathway in conjunction with antioxidation.

Aside from the anti-inflammation activities of AGN simulated by the DA in DSS-IBD model above, we reviewed in 2022 [1] other anti-infectious and anti-inflammatory activities of DA against sepsis induced by a lethal dose of methicillin-resistant Staphylococcus aureus (MRSA) [56] as pre-treatment (*ip* every other day for 0.4, 2 and 10 mg/kg doses). This study showed dose-dependent decrease of mouse mortality and the bacteremia, and attenuation of the cytokine storm at the highest dose. An even earlier study with DOH [57] in a prophylaxis setting by *ip* (1 ~ 100 mg/kg) before induction of experimental sepsis in mice exerted a dose-dependent reduction of sepsis lethality (ED_50_ ~ 10 mg/kg) induced by either lipopolysaccharide (LPS)/D-galactosamine (GalN) or high-dose LPS (20 mg/kg). In the post-infection therapy setting, DOH was injected *ip* twice at 2 h and 4 h after cecal ligation and puncture (CLP) microbial exposure. A plateau of therapeutic benefit was shown at 10 mg/kg and above. D was found to have a direct bactericidal activity with minimal inhibitory concentration ~ 12.5 mg/L (38 µM) against the most sensitive bacterial strain [58] and independently confirmed by a Thailand group with MIC ~ 32 mg/L (98 µM) [59]. Given the *ip* delivery of D and DA or DOH and *ip* exposure to pathogen microbes in some of these sepsis models, a direct bactericidal activity of D and DA was possible in the same *ip* space to decrease the microbial germ burden. Based on the known PK and metabolism of D/DA (to DOH) and nodakenin (to nodakenetin) as discussed earlier, systematic comparison of D, DA (and DOH) and nodakenin (and nodakenetin) with AGN and combination of these purified compounds in their relative abundance in AGN should be carried out in IBD and infection models to rigorously delineate the active chemicals and their relative contributions.

### Osteoporosis and Osteoarthritis and Joint Health

The beneficial impacts of AGN root powder supplement or D to inhibit bone loss in ovariectomized (OVX) rodent menopausal model or bacterial toxin-inflammatory cytokine LPS-induced bone loss have been thoroughly reviewed by us in 2022 [1]. Progress has been reported since for AGN extract in osteoarthritis (OA) models and nodakenin to ameliorate OVX-osteoporosis [15]. In surgically-induced OA by destabilization of medial meniscus (DMM) in male C57B mice [60], D treatment (daily gavage, 20 mg/kg, 8 weeks) after DMM attenuated the destruction of articular cartilage (severity score and subchondral cortical bone thickness) and decreased the serum inflammatory factor (IL-1β, IL-6 and TNF-α) levels to below the sham-operated control levels. A 2024 paper evaluated the potential of AGN in pain relief, functional improvement, and cartilage erosion delay using monosodium iodoacetate-induced OA rats and acetic acid-induced writhing mice [61], along with its anti-inflammatory effects on multiple targets in the serum and cartilage of these *in vivo* models. The animal experiments demonstrated significant analgesic and chondroprotective effects of AGN, along with functional recovery. AGN dose-dependently modulated inflammatory OA pathology-related proteins, including IL-1β, TNFα, MMP-13, and COX-2.

Using network pharmacology, molecular docking and dynamic simulation, a group in China explored potential targets and pathways of nodakenin in mitigating osteoporosis [15]. In cell culture exposure, they found that nodakenin promoted osteogenic differentiation of bone marrow stem cells (BMSCs) while activating the PI3K/AKT/mTOR signaling pathway as indicated by alkaline phosphatase activity and other osteogenic markers. An inhibitor of PI3K LY294002 reversed the signaling changes and inhibited the osteogenic differentiation effect of nodakenin. Nodakenin inhibited RANKL-induced osteoclastogenesis in the cell culture model. Oral nodakenin elevated mouse bone mass and ameliorated OVX-induced bone microarchitectural disorders. Their data suggest that nodakenin attenuated OVX-induced bone loss by enhancing osteogenesis and inhibiting osteoclastogenesis. Systematic comparison of D, DA (and DOH) and nodakenin (and nodakenetin) with AGN and combination of these purified compounds in their relative abundance in AGN should be carried out in OVX-osteoporosis and the different OA animal models for SAR to delineate the *in vivo* active compounds.

### Hematopoiesis Activity of AGN and Contribution from Nodakenin

Several recent studies have found beneficial hematopoiesis activities of AGN under a chemotherapy-induced myelosuppression context. Myelosuppression is a major adverse effect of many chemotherapy or radiation treatments. In the first study [62], the effects of AGN ethanol extract on myelosuppression were investigated in cyclophosphamide (CP)-treated aging mice (30 weeks old). AGN was administered *ip* at 10 or 20 mg/kg daily for 2 weeks, starting 3 days after CP injection. The AGN treatment significantly decreased CP-induced white blood cell levels while increased red blood cell and platelet levels in the peripheral blood and inhibited thymus and spleen atrophy in the CP-mice. AGN treatment also enhanced serum levels of interleukin (IL)−6 and tumor necrosis factor (TNF)-α. qRT-PCR results showed that AGN treatment decreased the mRNA level of IL-1b and stem cell factor (SCF) in the bone marrow (BM) and increased the mRNA expression of IL-3 and IL-6 in the spleen. AGN increased BM cells in the femur while decreasing apoptotic BM cells in the CP-mice. The authors verified D and DA content in the AGN extract, but did not test them as potential active chemicals for hematopoiesis.

A different group investigated the immune-enhancement effects of AGN extract and its yeast-fermented extract (FAN) in CP-induced immunosuppressed mice [63]. AGN increased the protein level of inducible nitric oxide synthase (iNOS) and the production of nitric oxide (NO) and immune-related cytokines in mouse splenocytes. AGN also restored CP-induced suppression of NK cell activity and splenocyte proliferation. Furthermore, AGN activated the ERK and p38 MAPK/NF-κB signaling pathways in mouse splenocytes via phosphorylation.

Yet another group of researchers used the CP-mice model along with cell culture studies to address nodakenin as a potential active compound for mitigating CP-induced myelosuppression [17]. They compared a mixture (SH003) of AGN, *Astragalus membranaceus* (Fisch.) Bunge, and *Trichosanthes Kirilowii* (Maxim.) with nodakenin. SH003 or nodakenin increased the levels of IFN-γ, IL-12, IL-2, IL-6, and TNF-α in the serum and spleen of CP-treated mice, alleviating CP-immunosuppression metrics. Their results from cell line culture and primary splenocytes suggested that SH003 relieved CP-immunosuppression through the activation of macrophages, splenocytes, and NK cells and that nodakenin contributed significantly to these activities. A systematic comparison of nodakenin (and nodakenetin) and D, DA (and DOH) at each’s relative abundance in the AGN extract and their combination in CP-mice or other myelosuppression models should be carried out to rigorously delineate the active chemicals.

## Topical Application of AGN Extract and D for Promoting Hair Growth

Diverging from the oral herbal supplement route, a Korean group studied AGN ethanol extract and equi-molar D for their impacts on hair growth in male mice through topical application [32]. Their prepared AGN ethanol extract contained 7.3% D. After the hair on mouse dorsal skin was shaved, the male mice received distilled water as control group, vs. 0.15% D, and 2% AGN root extract (equivalent to 0.15% D) diluted in distilled water topically twice a day in the treatment groups for 17 days. The results showed that the topical application of D and AGN extract promoted hair growth in equal efficacy. Hair growth was visibly facilitated from 7 days after the treatments began, and was complete at 17 days when the experiment was terminated, whereas the control group achieved below 50% hair coverage. The protein levels for inflammatory cytokines TNF-α and IL-1β (Western blot and IHC detections) in the dorsal skin samples were lower, and anti-inflammatory cytokines IL-4 and IL-13 were higher than those in the control group. High-mobility group box 1 protein, an inflammatory mediator and hair growth enhancer through prostaglandin E2, was elevated by the topical application of D and AGN extract than control. The authors suggested that AGN root extract and D as useful materials for developing hair growth-facilitating treatments, by counter regulating pro- and/or anti-inflammatory cytokines.

Given the topical application of AGN extract and D with equal efficacy and topical transdermal local delivery of D to hair follicles where androgen receptor (AR) is involved in driving male pattern alopecia [64], an alternative interpretation is the potent AR antagonist/degrader activity of D and DA in AGN discovered by us [4, 5] might have also contributed to the hair regrowth through repressing AR signaling. Further testing for hair growth promotion in female mice and orchiectomized male mice is warranted to shed light on whether sex affects the response and likely mechanisms.

## Molecular Targets for Active Chemicals

The updated *in vivo* bioactivities of AGN alcoholic extracts or root powder (Fig. 3) in diverse health domains, in aggregate, support D and DA, as the likely major “pro-drugs” for many of these benefits in extra-hepatic organs where their hepatic metabolite DOH predominates, while in the liver D and DA co-exist with DOH, and in GI lumen D and DA predominate (Fig. 2). A growing literature suggests that nodakenin (possibly with its aglycone and *in vivo* metabolite nodakenetin) may also contribute meaningfully to lipid metabolism [12], memory [13], anti-cancer [14], bone health [15], anti-inflammatory [16] activities as well as hematopoiesis [17]. The desired experimental design for future mechanistic research is a systematic comparison of an AGN supplement preparation with these compounds singly or in combination at each’s relative abundance in that supplement in relevant animal models to account for the efficacy outcomes. As these pyranocoumarin and furanocoumarin chemicals make up approximately one half of a typical AGN ethanolic extract, one should always be open minded to the poly-pharmacology possibility of additional active chemicals or their interactions with D, DA and nodakenin to fully account for a given bioactivity of that supplement.

The PK and metabolism knowledges (Sect. "Current Knowledge of Pharmacokinetics and Metabolism of Major Pyranocoumarins and Nodakenin") of D/DA → DOH and nodakenin *in vivo* conversion to its aglycone nodakenetin necessitate a re-assessment and re-appreciation of molecular targets by the lens of their relative peripheral circulating *C*_*max*_ and *AUC.* Given the estimated > 98% hepatic first pass conversion of D/DA → DOH, the molecular targets of DOH should therefore be of heightened emphasis for the non-hepatic, non-GI organs (Fig. 2). The following will discuss reported “direct” protein targets for DOH, D and DA and nodakenin. Figure 4 schematically highlights multifaceted connections among these proteins and their pathways to neurocognitive, pain relief, anti-cancer and metabolic benefits.

### Acetylcholine Esterase

History-wise, DOH was identified by Kang *et al*. as an AChE inhibitor more than two decades ago [7] based on the rationale that the neurotransmitter acetylcholine (ACh) was lower in brain regions of Alzheimer’s patients than age-matched healthy brain. They showed that DOH was the most potent AChE inhibitor out of 12 tested coumarins prepared from AGN (summarized in Table I) with IC_50_ for DOH of 28 µM vs. 390 µM for D (DA was not tested) and nodakenin was 68 µM, respectively [7]. AChE is expressed on the post-synaptic cell surface to effectively degrade ACh in the synaptic clefts following it release from presynaptic membrane fusion with its secretory vesicular granules. AChE inhibitors prolong the synaptic signaling in CNS to improve memory neuroplasticity. In addition to the memory benefit that could be attributable to a slowed degradation of ACh in CNS synaptic clefts by DOH (Fig. 4), the pain relief action observed for AGN and DOH might also be attributable to improved cholinergic signaling at the interneurons of the descending pain modulatory pathways in the CNS and brain stem region [65, 66]. Noteworthy is a 2023 study that has elucidated a novel cholinergic signaling circuit between the ventrolateral periaqueductal gray (vlPAG) and pedunculopontine tegmentum (PPTg) [66]. The authors used biosensor assays to reveal that acute and chronic pain states decreased ACh release in vlPAG and an inverse relationship of ACh release levels to nocifensive behavior activities. Activation of cholinergic projections from PPTg to vlPAG relieved pain through ⍺7 nicotinic ACh receptor (nAChR). Activating ⍺7nAChR with agonists or stimulating endogenous ACh inhibited vlPAG GABA^+^-neuronal activity (de-inhibition) through Ca^2+^ and peroxisome proliferator-activated receptor α (PPAR⍺)-dependent signaling. Beside the memory and pain relief benefits, it could be anticipated that peripheral cholinergic signaling improvements such as parasympathetic muscarinic cholinergic actions might benefit dry mouth (saliva secretion) and dry eye (tear secretion) issues by taking an AGN supplement.

### Androgen Receptor (AR) and Estrogen Receptor (ERα)

We have identified a weak partial agonist activity of DOH for androgen receptor (AR) [5] and estrogen receptor alpha (ERα) [6] in responsive cancer cell culture models, whereas D and DA acted as novel AR and ERα antagonists/degraders and apoptosis inducers [4, 5, 6]. The partial agonist action of DOH in extra-hepatic tissues including bone and muscle, adipose tissues on AR and ERα in males and females under respective hypogonadic state (androgen deprivation therapy for prostate cancer patients or menopause in women) may counter balance the hepatic D and DA actions on these receptors to contribute to the overall observed metabolic benefits of lowering hyperlipidemia and hyperglycemia reported in the rodent models [47, 48, 49] and lowering triglyceride and VLDL-cholesterol in a human clinical trial [53]. It is also possible that the reported hair growth promotion by topical application of AGN extract or an equimolar D could involve their potent suppression of AR signaling in hair follicles, as discussed earlier (Sect. "Topical Application of AGN Extract and D for Promoting Hair Growth").

### Monoamine Oxidase (MAO)-A

Monoamine neurotransmitters are biochemical messengers that regulate a variety of functions in the CNS, including: emotion, learning, motor control, sleep, wakefulness, consciousness, cognition, and attention. The most prevalent monoamine neurotransmitters are tryptophan-derived serotonin and tyrosine-derived dopamine and norepinephrine/noradrenaline [67]. The MAO-A and MAO-B isoforms are both located on the mitochondrial outer membrane and yield reactive oxygen species (ROS) and ammonia in the reaction with the monoamine neurotransmitters (X-CH_2_-NH_2_ + H_2_O + O_2_ + FAD → X-CO–H + H_2_O_2_ + NH_3_ + FADH_2_), leading to neuro-inactive products. Therefore, MAOs attenuate neurotransmitter levels and fluxes in the CNS and PNS along with vesicular monoamine transporters (vMATs) and their respective synthesis enzyme cascades. MAO-A is selective for serotonin and noradrenaline and is expressed in presynaptic neurons in CNS, liver hepatocytes and GI tract, adipose, thyroid and bladder [68]. MAO-B favors phenylethylamine and benzylamine as substrate, and is expressed in brain non-neuronal astrocytes/glial cells and PNS and is increased with aging. Tyramine and dopamine are mixed substrates for both MAO-A and MAO-B. In germline knockout mice, MAO-A-KO displayed aggression and autistic phenotypes that were responding to early post-natal blocking of serotonin signaling; whereas MAO-B-KO phenotype is not dramatic (non-aggressive) and not sensitive to a neurotoxin that causes dopamine-deficit Parkinsonism [63].

In a search for new monoamine oxidase (MAO) inhibitors, Lee *et al*. tested five coumarin derivatives and eight flavonoids and found D was a highly potent and selective inhibitor for human MAO-A (*IC*_*50*_ = 1.89 μM) and a reversible and competitive inhibitor (*K*_*i*_ = 0.17 μM), and that DA was only moderately inhibitory (*IC*_*50*_ = 12.8 μM), whereas DOH failed the initial screen at 10 μM [9] (Table I). Regarding MAO isoform selectivity, when screened at 10 μM, D, DA and DOH all lacked any inhibitory activity on MAO-B. In oncology, MAO-A is especially highly expressed in prostate cancer and correlated with Gleason grade and cancer aggressiveness [67, 69, 70].

MAO-A has been investigated for more than a decade for oncogenic and metastatic properties and therapeutic targeting by repurposing MAO inhibitors, with promising outcome for advanced castration resistant prostate cancer (CRPC) [71]. Given the expression of MAO-A in liver and GI tract where high levels of D and DA coexist with DOH (Fig. 2), MAO-A inhibition by D or DA could also have contributed to the metabolic benefits of AGN summarized in Fig. 3. One possible connection to cancer interception is the reduction of MAO-A mediated ROS [63] (Fig. 4). For pain relief, the role of MAO inhibitors to modulate neurotransmission in brain interneurons over descending pain modulatory pathway has been well known [72]. It could also be likely that AGN could improve other neurodegenerative conditions such as Parkinson’s through the slowed breakdown of dopamine and other monoamine neurotransmitters through MAO-A inhibition.

### Glutamic Acid Decarboxylase (GAD) and GABA_A_ Axis

With glutamate as substrate, GAD is the enzyme that catalyzes the synthesis of the inhibitory neurotransmitter gamma-aminobutyric acid (GABA) that slows brain activity and produces a calming effect (such as sleep and sedation) [73, 74]. Two isoforms of GAD are expressed in different organs. GAD65 is expressed in CNS for GABAergic neurotransmission, and therefore is found at nerve terminals and synapses. GAD67 synthesizes GABA for activities unrelated to neurotransmission, such as synaptogenesis and protection from neuronal injury. This latter function requires widespread, ubiquitous (basal tuning effect) presence of GABA. GAD is expressed also in pancreatic β-cells and auto-immune antibodies to these isoforms have been causally linked to type 1 diabetes due to destruction of insulin producing endocrine cells [75, 76].

The GABA_A_ receptors are ligand-gated ion channels, also known as ionotropic receptors. When GABA binds to a GABA_A_ receptor, it opens an ion channel that allows chloride ions to flow into the neuron causing its hyperpolarization and less likely to fire an action potential. GABA_A_ receptors are involved in a variety of behavioral and physiological effects, including feeding, cardiovascular regulation, and anticonvulsive activity. In the sleep study reviewed in Sect. "Additional Neuro-psycho-behavioral Benefits of Pyranocoumarins Suggest Multiple Molecular Targets", at the molecular/receptor level, DA (0.001, 0.01 and 0.1 µg/ml ≈ 0.003, 0.03 and 0.3 µM) increased intracellular Cl^−^ influx level in cultured rat hypothalamic primary neuronal cells [8]. In addition, DA exposure of these neuronal cells for 1 h increased the protein expression of not only GAD_65/67_ but also the GABA_A_ receptor subunits. D and DOH were not tested in the paper. Based on metabolism and PK knowledge of D and DA, DOH might have contributed to improving sleep, sedation and locomotor decline (ataxia) and should be tested side-by-side to address this prediction. The high potency of DA to trigger GABA_A_ ion channel axis lends credence to its probable target engagement *in vivo*. As epilepsy is triggered by excessive glutamatergic transmissions, which normally account for majority of cerebral cortical energy-intense neuronal activities, the increased GAD_65/67_ expression by DA or D in the neuronal cells might have contributed to dampening of glutamatergic firings in the observed anti-epilepsy action of AGN [40].

### Glutamate Dehydrogenase (GDH or GLUD)

A 2023 publication reported identification of GDH as a target for D and DA through computational simulation which predicted their stronger binding than tea polyphenol inhibitor EGCG [10]. The authors experimentally confirmed *IC*_*50*_ for D and DA ~ 1.0 μM and ~ 1.4 μM, respectively [10] (Table I). Unfortunately, they did not test DOH for the GDH inhibitory potency. With respect to a role in malignancy, overexpression of GDH1 had been causally linked to driving prostate cancer growth and aggressiveness [77]. In addition, cancer addiction to glutamine and glutaminolysis to drive up glutamate fluxes to meet cancer cellular energetics demand, and to supply intermediates for membrane lipids, purine biosynthesis in hypoxic tumor environment is now a recognized metabolic hallmark in solid malignancies including prostate cancer and a metabolic vulnerability for innovative cancer interception [78] (Fig. 4).

Two isoforms of human GDH are encoded by two genes, GLUD1 and GLUD2, which are located on chromosomes 10 and X, respectively [79, 80]. GDH2 is only found in human and greater apes and is expressed in brain neurons and astrocytes, and in the testes and other steroidal hormone de novo synthesis organs such as adrenal glands, but GDH2 is not expressed in the liver, in which GAH1 is a prominent mitochondrial matrix protein (~ 10% of liver matrix protein mass). GDH1 and GDH2 are each composed of 6 homomeric subunits of ~ 500 amino acids. As highlighted in Fig. 4, GDH catalyzes the oxidative deamination of L-glutamate to α-ketoglutarate (TCA cycle intermediate, leading to increased lipogenesis substrate citrate) using NAD(P) + as a coenzyme, generating reducing metabolites NAD(P)H for anti-oxidation and de novo lipogenesis and other biosynthetic reactions. Being a major liver enzyme (~ 1% protein mass), GDH1 serves as a central hub for metabolic coordination among amino acid fluxes, cellular energetics and lipogenesis. A classic enzyme extensively investigated for allosteric controls, GDH1 is regulated by a wide array of metabolites/ligands. The high-energy signal molecule GTP from TCA cycle is a strong inhibitor by increasing the GDH1 binding affinity for its own reaction product α-ketoglutarate which is a key TCA intermediate, while ATP is a weaker inhibitor (~ 100-fold less than GTP), whereas ADP is an activator of GDH1 in contrast to GTP and ATP as inhibitors. Lipogenesis and steroidogenesis intermediate or end products palmitoyl CoA and steroid hormones, and estrogen receptor agonist diethylstilbestrol (DES) are potent hydrophobic inhibitors, as is the tea polyphenol EGCG. On the other hand, the branch chain aliphatic amino acid leucine (the most abundant AA in proteins and the best indicator of protein intake status) is a potent allosteric activator of GDH1. GAH1 is also expressed in pancreatic endocrine β-cells, regulating insulin release per K^+^-ATP-Ca^2+^ flux responding to the higher ATP/ADP ratio after feeding. Certain mutations in GDH1 had been identified to cause a hypoglycemic disorder called hyperinsulinism-hyperammonemia syndrome due to the loss of ATP inhibition. Therefore, the GDH1 activity is finely tuned by the balance of, and interplay among, many allosteric inhibitors and activators to fulfill its central coordinating hub role in the liver among amino acid fluxes, cellular energetics and lipogenesis.

The GDH2 form is only found in humans and greater apes, likely an evolutional advantage to their increased brain size compared to other mammals [81]. The intron-less GDH2 arose from retro-position of GDH1 gene some 23 million years ago in the ancestral humanoid primates. The human GDH2 is X-linked and found mainly in brain pyramidal neurons and testicular tissue where high lactate is amenable to supply the excessive ATP need in preference over glucose. Due to evolutional pressure, GDH2 mutations from GDH1 rendered this neuron and testicular form less sensitive to GTP inhibition but more sensitive to ADP activation, less thermostable (befitting lower temperature in postnatal testes), and having a lower basal activity than GDH1 and is super-activatable by ADP (greater dynamic range). From sequencing GDH1 and GDH2 genes in Parkinson’s disease patients, an A445S mutation in GDH2 that increased enzyme activity was found to be correlated with 6–13 year earlier onset of symptoms (excessive ammonia toxicity on pyramidal neurons), but not in females likely due to the strong estrogen inhibition of the mutant GDH2. Brain-specific transgenic animals expressing human GDH2 in the pyramidal neurons (under the human promoter) in the hippocampal regions showed improved memory benefits and greater neuronal plasticity than wild type mice [82].

As liver GDH1 serves as a central hepatic control hub integrating numerous allosteric control signals in regulating amino acid fluxes, cellular energy charge status, lipogenesis (adipose) and steroidal genesis, the reported metabolic syndrome modulating effects of AGN in rodent models [47, 48, 49] and lowering of triglyceride and VLDL-cholesterol in a human clinical trial [53] may be attributable to the portal delivery of D and DA to liver and their pharmacodynamic interactions in the hepatocytes (Fig. 4). As glutamatergic neurons account for the majority of cortical CNS synaptic firings/connections and are extremely energy demanding, the inhibitory action imposed by D and DA on GDH and their promoting action on GAD/GABA_A_ axes may collectively shunt glutamate toward sedation and anxiolysis. Whether GDH2 in brain neurons is significantly affected by high circulating levels of DOH to contribute to the reported neuro-cognitive benefits remains unanswered at the moment.

### Transient Receptor Potential Vanilloid 1 (TRPV1)

TRPV1 is an ion channel that detects harmful stimuli like heat and capsaicin, the pungent ingredient in chili peppers. TRPV1 is mainly expressed in PNS for nociception. TRPV1 is also expressed in non-neuronal cells, such as: epithelium of bladder and lungs, hair cells of the cochlea, mast cells, glial cells, bronchial epithelial cells, uroepithelial cells and keratinocytes in the skin. In the paclitaxel-CINP mouse model study [11], the authors used a somatic cell hybrid of a rat embryonic dorsal root ganglion [DRG] and mouse neuroblastoma cell line N18TG (F11 cell culture) to test the direct impact of D on capsaicin-TRPV1-intracellular Ca^2+^ rise with IC_50_ ~ 1 µM. The structure activity relationship (SAR) study should be applied to the *in vitro* tests for DOH and DA. A direct *it* injection of DOH and DA should be tested in the same *i.t.* treatment model to address whether the pain-killing action was mediated by DOH or other D or DA-mediated mechanism. TRPV1 expression has been examined in cancers of many organ sites and established cancer cell lines [83]. There are many confusing cell culture studies of TRPV1 agonists as well as antagonists regarding cancer cell death and proliferation.

### DOH Binding Proteins

More than a decade ago, Kang *et al*. have identified DOH binding proteins from a mouse brain cell line by affinity chromatography and mass spectrometry [84]. These included intracellular proteins heat shock protein 90 kDa beta member 1 (GRP94 or HSP90B1), enolase 1 (ENO1), actin gamma 1 (ACTG1), heterogeneous nuclear ribonucleoprotein A2/B1 (HNRNPA2B1) and non-muscle myosin heavy polypeptide 9 (MYH9). Further validation of their binding affinities and *in vivo* relevance have not been reported. A common feature of many of these proteins is possession of ATP binding domain, inspiring us to screen for protein kinases (kinome) which use ATP to phosphorylate target proteins to alter their enzymatic or functional activities.

### Rho-associated Protein Kinase 1 and 2

(ROCK1/2) as potential targets for DA and DOH (see next Sect. "*In vitro* Screening for Direct Protein Kinase Targets for DOH and DA").

## *In Vitro* Screening for Direct Protein Kinase Targets for DOH and DA

### Chemicals and Reagents

DOH was purchased from Aktin Chemicals, Inc. (Chengdu, P.R. China; Website: www.aktinchem.com) per contract purification. The purity was verified to be higher than 98% by HPLC, ^1^H-NMR, and ^13^C-NMR. Ethyl acetate, PEG 400, Tween 80 were purchased from Sigma-Aldrich Company (St. Louis, MO). DA was purchased from Cayman Chemicals.

### *In Vitro* Kinome Screening Platforms

Two distinct platforms were used for initial screening with 50 µM DOH vs. DMSO vehicle. The first was the Eurofins DiscoverX KINOMEscan® (Freemont, CA USA) profiling platform, which employed a proprietary active site-directed competition binding assay to quantitatively measure interactions between a test compound (i.e., DOH) and 468 human protein kinases and disease relevant mutant variants either directly (sterically) or indirectly (allosterically) to prevent a kinase binding to its immobilized substrate (ligand). The top 8 hits were subjected to secondary screening verification by DOH concentration-titration for dissociation constant (*K*_*d*_). For ROCK1 and ROCK2, DA was chosen for *K*_*d*_ titration as well, because it is ~ 10 × higher than D in peripheral circulation due to its resistance to hydrolysis by carboxyesterase-2 (Fig. 1C).

The second screening was the Reaction Biology® (Malvern, PA) kinase activity inhibition tests, which screened for more than 600 protein kinases and lipid kinases, using miniaturized radioisotope-based assay platform with γ-^33^P-ATP, followed by confirmation with *IC*_*50*_ kinase profiling service with 5 concentrations of test compound (i.e., DOH and DA) with curve fitting.

## Results

### DOH Competed for Binding to Substrate Binding Site of ROCK1/2

Initial screening at 50 μM DOH identified hits that competed for binding (Supplement Table S1). On verification titration testing, ROCK1, ROCK2 (Table I), DRAK2, and MKNK2 of the top 8 hits were confirmed to compete for the substrate binding with *K*_*d*_ ranging from 8 to 23 μM (Table S1). However, ERK4, LKB1, CDK9, CDC2L1 were “false positives”, failing the validation titration testing. *Kd* titration for DA revealed lower values for ROCK1 and ROCK2 (Table I), therefore, stronger binding than DOH.

### DA and DOH Inhibited the Activity of ROCK1/2

The Reaction Biology® screening at 50 μM DOH showed a weak inhibition of ROCK1 and 2 activity by 27% and 43%, respectively (Supplement Table S2). Reassuringly, the activity screening for LKB1 and CDK9, which were “false positive” per DiscoverX verification, showed no inhibition by DOH at 50 μM on their activity. On verification titration profiling, the *IC*_*50*_ for ROCK 1 and ROCK 2 was 81 and 85 µM, respectively (Table I). The *IC*_*50*_ for DA was ~ ¼ of DOH (Table I), therefore ~ 4 × more potent than DOH. In spite of the modest inhibitory potency of DOH and DA, and likely D as well, in the single to double digit micromolar range, the selectivity was considerable out of > 400 protein kinases screened by both platforms. In mouse and rat models, double digit micromolar DOH was achievable in the blood with the oral gavage dosing of AGN or D/DA [26, 85, 86]. Given preservation of D and DA in the gastrointestinal lumen and portal vein system draining into the liver for first-pass metabolism (Fig. 2), they could have contributed directly to inhibit ROCK1/2 in these organs and their diseased conditions, such as liver steatohepatitis [87, 88] and IBD [69].

## Discussion and Integration

ROCK1 is mainly expressed in non-neuronal tissues and ROCK2 is expressed abundantly in brain neurons and spinal cord [89]. Increasing with age, ROCK2 activation has been linked to neuronal cell death in Alzheimer’s brain and other neurodegenerative diseases [90, 91] and might be a potential therapeutic target to retard the progressive neuronal loss [92]. In brain neurons, ROCK2 plays a significant role in regulating the structure and stability of dendrites (the branches of a neuron that receive signals) and dendritic spines through modifying actin cytoskeletons. With respect to cardiovascular functions, activated ROCK plays a pivotal role in processes leading to cardiovascular diseases such as general hypertension, pulmonary arterial hypertension (PAH), atherosclerosis, vasospastic angina pectoris, stroke, diabetes, cardiac ischemia/reperfusion (I/R) injury and heart failure [93]. Isoform-specific knockout mouse models revealed ROCK1 contributing to the pathogenesis of cardiac fibrosis and ROCK 2 in cardiac hypertrophy, respectively [93]. In addition to neurons and glia, ROCK affects many other cell types in the brain such as vascular endothelial cells, smooth muscle cells, immune and inflammatory cells etc. in terms of cell contraction and motility. These processes are related to the pathophysiology of cerebral ischemia stroke and other brain injuries [94]. A recent study reported ROCK 1/2, and inflammatory cytokines (IL-1 and IL-6) were upregulated in vascular dementia models [95] while the expression of claudin-5, which maintains the blood–brain barrier, and MAP2 as a nerve cell-specific factor, were decreased in the hippocampal region. Thus, ROCK pathway activation loosened the tight junction of the blood–brain barrier and increased the influx of inflammatory cytokines into the hippocampal region, leading to neuronal death and cognitive dysfunctions. The DOH and DA targeting of ROCK1/2 as revealed by our kinome screenings could therefore contribute to the ischemic stroke prophylactics and memory benefits that were reported for AGN (Fig. 3).

ROCK 1 has been found to increase in patients with nonalcoholic fatty liver disease (NAFLD) or nonalcoholic steatohepatitis (NASH) and other liver disorders [87, 88]. The earlier work by Huang *et al*. initially demonstrated that ROCK1 knockout in mouse NAFLD model and genetic obesity mouse model ameliorated the NAFLD and diabetes phenotype metrics [87]. They also found that the obesity drug metformin was able to down regulate the ROCK1/AMPK axis [87]. Similarly, hepatic ROCK1 expression levels and activity were increased in mice with NASH induced by a Western-type diet that is high in fat, fructose, and cholesterol (the FFC diet). Hepatocyte-specific ROCK1 knockout mice on the FFC diet decreased liver steatosis, hepatic cell death, liver inflammation, and fibrosis compared with wild type littermate FFC-fed controls and attenuated myeloid cell recruitment, which was secondary to the hepatocyte actions because myeloid cell-specific ROCK1 deletion did not affect NASH development in FFC-fed mice. Pharmacologically, a novel small molecule kinase inhibitor of ROCK1/2 that preferentially accumulated in liver tissue ameliorated insulin resistance and decreased liver injury, inflammation, and fibrosis in mice fed the FFC NASH diet. Given the portal vein hepatic first pass metabolism, the levels of D and DA prior to hydrolysis by Cyp 2C19, 3A4 and/or CES2 (Fig. 1C) could be high enough to directly inhibit liver ROCK1 to contribute to the anti-hyperlipidemia activity in the different animal models [87, 88] and in the human trial outcome [53].

ROCKs have been found to be involved in the pathogenesis of a variety of autoimmune diseases and a crucial role of ROCK2 in inflammatory bowel disease (IBD) has been reported [96]. These authors demonstrated that the levels of ROCK2, but not ROCK1, activity were significantly upregulated in peripheral blood mononuclear cells (PBMC) and inflamed mucosa from IBD patients using a ROCK activity assay, and that ROCK2 activity in intestinal mucosa was positively correlated with disease severity. Stimulation with TNFα markedly upregulated ROCK2 activity in IBD CD4 + T cells through NF-κB signaling. Blockade of ROCK2 activity using Slx-2119 inhibitor drug suppressed proinflammatory cytokines in inflamed mucosa from IBD patients including TNFα blocking infliximab-unresponsive CD patients, and inhibited IBD CD4 + T cells to differentiate into Th1 and Th17 cells through downregulating phosphorylated Stat1 and Stat3, but promoted T_reg_ cell differentiation through upregulating phosphorylated Stat5. In a mouse model, oral Slx-2119 markedly ameliorated intestinal mucosal inflammation in TNBS-induced colitis and decreased proinflammatory cytokines productions in inflamed colon. Their data support that ROCK2 plays a critical role in inducing mucosal T cell activation and inflammatory responses in IBD. Since D and DA stay intact in the gastrointestinal lumen and mesentery vessels draining into the hepatic portal vein system (Fig. 2), they may directly inhibit ROCK2 activity on site to contribute to their (and AGN’s) observed ameliorating actions on IBD models.

In osteoarthritis (OA), studies have shown activation of ROCK signaling is involved in early phase response to abnormal mechanical stimuli [97]. ROCK interacts with OA pathological factors and induces cartilage degeneration through the degradation of chondrocyte extracellular matrix (ECM). It was demonstrated that the adipokine nesfatin-1 improved cytoskeleton integrity manifested as higher F-actin/G-actin ratio and more organized actin fiber structure [98]. Mechanistically, RhoA activator and inhibitor studies revealed a regulation of autophagy and cytoskeleton integrity by nesfatin-1 via RhoA/ROCK pathway. Nesfatin-1 significantly ameliorated IL-1β induced cartilage degeneration in the destabilization of the medial meniscus (DMM) model [98].

In the oncology domain, ROCKs have been demonstrated to be over-expressed in many types of cancer including prostate cancer [99, 100]. In prostate cancer, the angiogenesis, motility invasion and metastasis promoting roles of ROCKs have been well established [101] (Fig. 5). It is also noteworthy of their interaction and activation of the c-Myc oncoprotein that is overexpressed in a high percentage of prostate cancer [102]. Our discovery of ROCK inhibitory activity of DA and DOH adds additional support for the polypharmacology tenet of multi-chemicals and multi-targets in the many health promoting and disease fighting benefit domains.

## Advocacy for Clinical Trial Study of AGN Supplement for Mitigating Androgen Deprivation Therapy Side Effects in Prostate Cancer Patients

According to the US National Cancer Institute official website [103], androgen deprivation therapy (ADT) has many side effects, including: loss of interest in sex (lowered libido), erectile dysfunction, hot flashes, loss of bone density, bone fractures, loss of muscle mass and physical strength, changes in blood lipids, insulin resistance, weight gain, mood swings, fatigue, growth of breast tissue (gynecomastia) in addition to the metastatic bone pain. These are grouped into different health domains: bone health, muscle and strength, sexual health, emotional health, weight and metabolism, skin (dry skin, rashes, and skin that becomes red, darker, or irritated), vasomotor functions (hot flashes, night sweats, headaches, itching) and GI health. The UpToDate website provides detailed description of each risk domain and management options [104]. A 2024 metanalysis based on 27 studies with a total of 2,543,483 patients, including 900,994 with prostate cancer who received ADT, 1,262,905 with prostate cancer who did not receive ADT, and 334,682 patients without prostate cancer who did not receive ADT [105] had revealed significantly increased Hazard Ratios (HR) of 1.66, p < 0.00001 for depression, HR 1.57, p < 0.00001 for Parkinson's disease, HR 1.20, p < 0.00001 for dementia, vascular dementia HR 1.30, p < 0.00001, and HR 1.26, p = 0.0007 for Alzheimer's disease.

Based on the documented *in vivo* bioactivities of AGN and D/DA, DOH and nodakenin (Fig. 3), we hypothesize that AGN supplement given at sufficient high yet safe dosage will mitigate some or all of the side effects of ADT, without compromising its efficacy to treat the prostate malignancy, given the well documented wild spectrum anti-cancer activities of AGN and its notable phytochemicals [1] (Fig. 3). Figure 5 stylizes the mechanistic connections of the beneficial actions of AGN or its phytochemicals to the relevant health domains. In particular with respect to vasomotor symptoms (VMS) and emotional health issues induced by ADT, our recently documented sedation and hypothermia effect of DOH [37] are noteworthy for anxiolysis and pyrolysis. Because ROCK 1/2 activation is involved in neuronal cell death in AD and other neurodegenerative diseases and neurovascular dysfunctions, our newly discovered inhibitory activity of DA and DOH on ROCK1/2 provided additional mechanistic links toward AGN’s multifaceted neuro-cognitive and vascular benefits.

Whereas the memory loss prevention, and lipid (triglycerides and VLDL-cholesterol) lowering activities of AGN extracts had been demonstrated in human clinical trials in Korea [39, 53] (albeit at supplement dose <  ~ 1000 mg per day, and small group size < 50 participants per group), rigorous trial design with greater, yet, safely tolerated AGN extract dosages would be anticipated to afford a higher probability of successfully assessing the efficacy of AGN to mitigate ADT side effects and improve the quality of life of the prostate cancer patients, especially in US and Western countries whereupon racial and genetic, diets and lifestyle and cultural differences from Korean subjects might affect acceptability and effectiveness of the AGN modality. Our team is planning a placebo-controlled trial in the US cancer patient population undergoing ADT and is anticipating synergistic gains from the AGN safety knowledge from our ongoing Phase I/II trial in prostate cancer patients (NCT06600698)[106].

## Conclusions and Perspective

More than a dozen *in vivo* medicinal activities of AGN root powder and alcoholic extracts and/or their pyranocoumarins and the furanocoumarin nodakenin highlight their polypharmacology nature of multiple chemicals and multiple molecular and pharmacodynamic targets. A critical appraisal of published molecular targets encompassed multiple regulators of neurotransmitter (acetylcholine, glutamate, GABA, select monoamines) levels and fluxes in CNS and PNS for neurocognition and pain relief, sex hormone receptor signaling, hepatic and extrahepatic energy and lipid, glucose and nucleic acid metabolisms. Our discovery of ROCK1/2 as novel targets for DA and DOH and integration with published activities of DOH, D and DA, nodakenin and knowledge of their PK metrics permitted a more holistic appreciation of their contributions to mediating the various beneficial bioactivities of AGN extracts. Elucidating the pertinent mechanisms of action for these *in vivo* benefits requires rigorous and systematic comparison of an AGN supplement preparation with these compounds singly or in combination at each’s relative abundance in that AGN supplement in relevant animal models. Team science and interdisciplinary and transdisciplinary collaborations with standardized herbal preparations and animal models are essential to yield actionable knowledge for more efficient and effective human translation studies on a global scale. Rigorous human clinical trials are advocated and timely for AGN extracts for managing and treating side effects of androgen deprivation therapy in prostate cancer patients, peri and post-menopause health challenges in women, prevention and slowing down neurodegenerative diseases in aging populations, cardiovascular dysfunctions and diseases, in addition to their oncology benefits.

## Supplementary Information

Below is the link to the electronic supplementary material.

Supplementary file1 (XLSX 21 KB)

Supplementary file2 (XLSX 22 KB)

## Data Availability

All experimental data generated or analyzed for kinome screening and validation during this study are included Table I and supplement Tables S1-S2.

## Abbreviations

AGN: *Angelica gigas* Nakai
AChE: Acetylcholinesterase
CES2: Carboxyesterase-2
Cyp: Cytochrome P450
D: Decursin
DA: Decursinol angelate
DOH: Decursinol
GAD: Glutamic acid decarboxylase
GABA: Gamma-aminobutyric acid
GDH/GLUD: Glutamate dehydrogenase
IBD: Inflammatory bowel disease
MAO-A: Monoamine oxidase type A
NASH: Non alcoholic steatohepatitis
NAFLD: No alcoholic fatty liver disease
ROCK: Rho-associated protein kinase
TRPV1: Transient receptor potential vanilloid 1; a.k.a. capsaicin receptor, vanilloid receptor
